# A single NaK channel conformation is not enough for non-selective ion conduction

**DOI:** 10.1038/s41467-018-03179-y

**Published:** 2018-02-19

**Authors:** Chaowei Shi, Yao He, Kitty Hendriks, Bert L. de Groot, Xiaoying Cai, Changlin Tian, Adam Lange, Han Sun

**Affiliations:** 1 Department of Molecular Biophysics, Leibniz-Forschungsinstitut für Molekulare Pharmakologie, 13125 Berlin, Germany; 2 Hefei National Laboratory for Physical Sciences at the Microscale, School of Life Sciences, University of Science and Technology of China, 230027 Hefei, P. R. China; 3 Biomolecular Dynamics Group, Max Planck Institute for Biophysical Chemistry, 37077 Göttingen, Germany; 4 Institut für Biologie, Humboldt-Universität zu Berlin, 10115 Berlin, Germany; 5 Section Structural Biology, Leibniz-Forschungsinstitut für Molekulare Pharmakologie, 13125 Berlin, Germany

## Abstract

NaK and other non-selective channels are able to conduct both sodium (Na^+^) and potassium (K^+^) with equally high efficiency. In contrast to previous crystallographic results, we show that the selectivity filter (SF) of NaK in native-like lipid membranes adopts two distinct conformations that are stabilized by either Na^+^ or K^+^ ions. The atomic differences of these conformations are resolved by solid-state NMR (ssNMR) spectroscopy and molecular dynamics (MD) simulations. Besides the canonical K^+^ permeation pathway, we identify a side entry ion-conduction pathway for Na^+^ permeation unique to NaK. Moreover, under otherwise identical conditions ssNMR spectra of the K^+^ selective NaK mutant (NaK2K) reveal only a single conformational state. Therefore, we propose that structural plasticity within the SF and the selection of these conformations by different ions are key molecular determinants for highly efficient conduction of different ions in non-selective cation channels.

## Abstract (web summary)

NaK is a non-selective cation channel that conducts sodium (Na^+^) and potassium (K^+^) equally well. Here authors use ssNMR and MD simulations to show that the selectivity filter of NaK adopts two conformations in the absence of ions, one of which is preferred by Na^+^ and the other by K^+^.

## Introduction

Sodium (Na^+^) and potassium (K^+^) are the most abundant monovalent cations in biological systems and play an essential role in the homeostasis and electrical activity of living cells. NaK belongs to the class of non-selective monovalent cation channels that conduct both K^+^ and Na^+^ ions with equal efficiency^1^. In contrast, K^+^ selective channels are characterized by the remarkable ability to select K^+^ over Na^+^ by a ratio greater than 1000:1^2^. This high selectivity in K^+^ channels is attributed to the narrow pore of the channel, known as the selectivity filter (SF). The conserved sequence TVGYG forms four K^+^-binding sites (S1–S4), which are composed from the backbone carbonyls and the threonine hydroxyl group^3^. The high conductance of K^+^ selective channels has been explained in terms of either a multi-ion “knock-on” mechanism^4–6^ or the recently proposed “direct knock-on” mechanism^7^. Although NaK has a similar SF sequence (TVGDG) as K^+^ selective channels, the mechanism by which NaK conducts both Na^+^ and K^+^ at extremely high rates remains under debate^8–10^.

High-resolution crystal structures of NaK revealed the conservation of only the S3 and S4 ion binding sites, with the upper part of the SF becoming a vestibule^11^. Notably, ion binding sites identical to those of NaK were observed in a recently solved cryo-electron microscopy structure of the biologically and physiologically important hyperpolarization-activated cyclic nucleotide-gated (HCN) channel^12^. HCN1 exhibits significant similarities to NaK in ion selectivity (K^+^/Na^+^ ~4), SF sequence, and structural architecture. These observations, together with mutagenesis studies on NaK, suggested that loss of contiguous binding sites (S1 and S2) is responsible for ion non-selectivity^13,14^. Moreover, no structural rearrangement was observed in the crystal structures of NaK with Na^+^, K^+^, or rubidium (Rb^+^) and a single SF conformation was detected for NaK in bicelles at high temperature by solution-state NMR^15^. The different ions were therefore proposed to permeate through the same SF conformation with different preferential binding sites^11,16^.

In this study, we use a combination of ssNMR and advanced molecular dynamics (MD) simulations to investigate the detailed mechanism of ion non-selectivity in the NaK channel. Distinct from other methods in structural biology, ssNMR makes it possible to study membrane proteins in native-like lipid bilayers at room temperature and under physiological buffer conditions^17–20^. The MD simulations allow us to determine the conductive conformation of NaK for Na^+^ and K^+^, and further enable us to investigate the detailed permeation mechanism of these ions^5–7^. Intriguingly, we identify two conformations of the SF in NaK, one of which is preferred by Na^+^ and the other by K^+^. We further underline the functional importance of the conformational preferences induced by Na^+^ or K^+^ binding by using MD based permeation simulations. These results not only provide valuable insights into the conduction mechanism of NaK, but also have important implications for our understanding of the selectivity difference between the K^+^ selective and non-selective channels.

## Results

### ssNMR measurements and sequential assignments of NaK

**Fig. 1:**
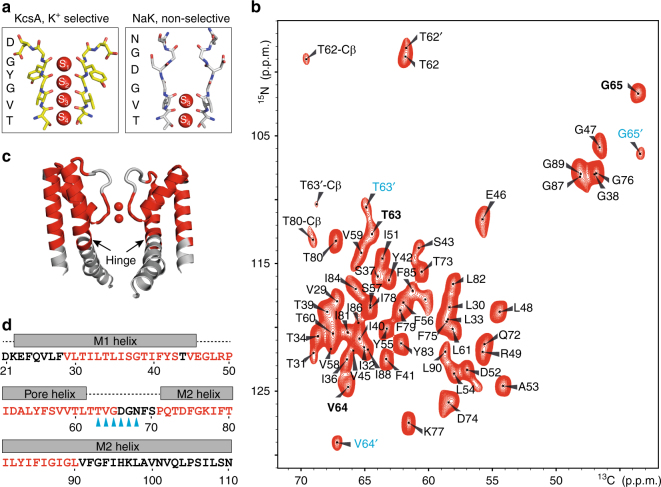
High-resolution ssNMR spectra of NaK. **a** SF in the crystal structure of KcsA (PDB ID: 1K4C) and NaK (PDB ID: 3E8H). **b** 2D NCA correlation spectrum of NaK in the presence of 50 mM K^+^ with the signal assignment. SF residues are indicated in bold and the ion-free state is labeled with a prime (′) and in cyan to differentiate it from the K^+^/Rb^+^-favored state (no prime; in black). **c** Crystal structure of the open state of NaK displaying two opposing subunits. The hinge region is marked with an arrow and assigned residues are indicated on the structure in red. **d** Amino acid sequence of NaK with secondary structure elements based on the crystal structure indicated above the sequence. Assigned residues are shown in red and SF residues are accentuated by cyan arrows

We first prepared four uniformly [^13^C, ^15^N]-labeled NaK samples in liposomes: (A) at low ionic conditions (<1 μM) or with 50 mM of (B) Na^+^, (C) K^+^, or (D) Rb^+^. Single-channel conductance measurements verified that NaK in our lipid bilayer preparation is functional (Supplementary Fig. 1). De novo sequential backbone and side chain chemical shift assignments were obtained from two-dimensional (2D) and three-dimensional (3D) correlation spectra^21^ of the Na^+^-containing sample (Supplementary Figs. 2, 3). All visible signals in the spectra could be assigned to 56 residues, which are highlighted in the crystal structure and amino acid sequence of NaK (Fig. 1). Unassigned residues cluster to two different regions: (i) D66-S70 in the vestibule and extracellular entrance of the SF and (ii) the N-terminus up to F28 and from V91 until the C-terminus below the hinge region in the M1 and M2 helices. The signals from these regions cannot be detected in dipolar-coupling-based experiments, most probably due to conformational heterogeneity caused by strong structural dynamics. The signals from flexible residues, as observed in scalar-coupling based experiments, strongly correlate with the residue types found in the C- and N-termini (Supplementary Fig. 4). However, the residues in the lower parts of the M1 and M2 helices are still not visible, implying that dynamics of these parts are relatively slow.

### Two distinct states of the SF detected by ssNMR

**Fig. 2:**
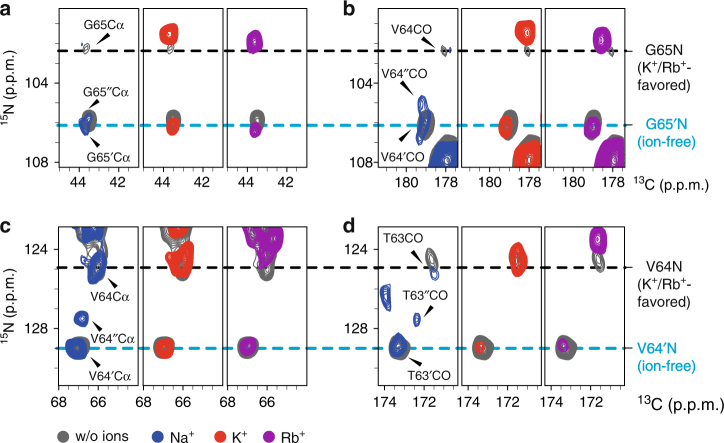
K^+^/Rb^+^-favored and ion-free state observed in ssNMR. Comparison of 2D NCA (**a**, **c**) and NCO (**b**, **d**) spectral closeups of SF residues in sample A (<1 µM ions; gray), B (50 mM Na^+^; blue), C (50 mM K^+^; red), and D (50 mM Rb^+^; purple)

The 2D ^15^N_i_-^13^Cα_i_ (NCA) and ^15^N_i_-^13^CO_i-1_ (NCO) correlation spectra of NaK at low ionic conditions showed two sets of signals for SF residues V64 and G65. Two sets of signals are also visible in the PDSD spectrum, highlighted for residue V64 in Supplementary Figure 5. The signal intensities suggest one dominant and one minor populated state, which are in very slow exchange compared to the μs-ms NMR timescale. For the upfield (lower chemical shift) nitrogen signals of V64 and G65, we observed ion-dependent chemical shift changes (low ionic vs K^+^ vs Rb^+^), while the downfield (higher chemical shift) nitrogen signals remained unaffected (Fig. 2). Since chemical shifts are highly sensitive to the local electronic environment induced by different ion types and occupancies in the SF^19,22^, this result strongly suggests that the upfield nitrogen signals correspond to a K^+^/Rb^+^-favored state and the downfield nitrogen signals correspond to an ion-free state.

The two detected states of the SF can be stabilized under different conditions. Low ionic conditions show the ion-free state as dominant, although the K^+^/Rb^+^-favored conformational state can still be observed. In contrast, this state becomes more dominant with increasing K^+^ concentration (50 mM: Fig. 2, 150 mM: Supplementary Fig. 6). Intriguingly, ssNMR spectra of NaK in complex with Na^+^ revealed a different equilibrium where the ion-free state is the major population even though ions are present. Furthermore, ssNMR spectra of the Na^+^ sample revealed increased structural plasticity in the SF by significant line broadening for residue G65 (nitrogen dimension in the NCO spectrum) and by the appearance of additional peaks for V64 (Fig. 2 and Supplementary Fig. 6). Under high Na^+^ (150 mM) conditions, the signal intensity of the SF residues decreases further and both G65 and V64 show even more additional peaks that portray undefined conformations.

### Structural characterization of the two SF states

**Fig. 3:**
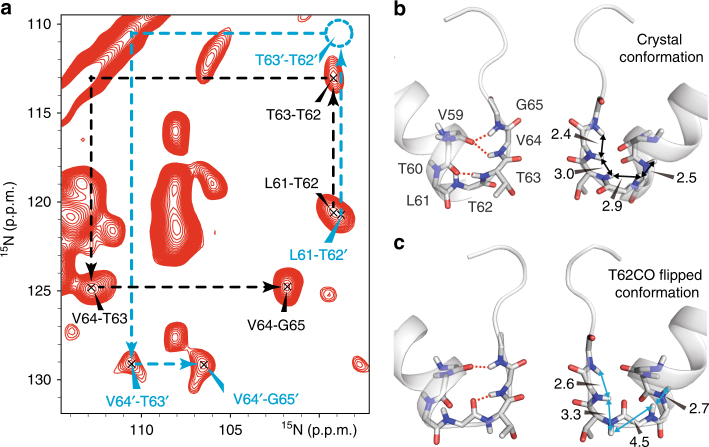
Crystal and T62CO flipped conformations. **a** NHHN spectrum of NaK with 50 mM K^+^ showing the ion-free (cyan) and K^+^/Rb^+^-favored (black) conformational states. The predicted position of the T63’-T62’ cross-peak is circled in dashed cyan. **b**, **c** Hydrogen bond network in the crystal (**b**) and the flipped (**c**) conformations of NaK. Distances (Å) between adjacent amide protons are indicated and hydrogen bonds are shown with red dashed lines

The nitrogen chemical shift differences between the ion-free and K^+^/Rb^+^-favored states were up to 4 p.p.m. for V64 and G65. Such large nitrogen chemical shift differences are usually related to structural rearrangements associated with changes in hydrogen bonding. Carbonyl flipping in the SF should perturb the hydrogen bonding network and carbonyl flipped conformers have been observed in several crystal structures and simulations of KcsA^6,23,24^. In order to investigate alternative stable conformations of the SF, we undertook MD simulations on channels where we generated a set of backbone carbonyl flipped conformers for T62 to G65. Only the T62 flipped carbonyl conformer in all four subunits (Fig. 3c) was stable during the simulations under the ion-free condition (only ions to neutralize the system were present in the simulation box). This flipped conformation removes two hydrogen bonds for T63NH-T60CO and V64NH-V59CO, but permits the formation of a new hydrogen bond for V64NH-T62CO.

SsNMR distance measurements on NaK with K^+^ provided strong support that the crystal structure and the flipped conformation correspond to the K^+^/Rb^+^-favored and ion-free states, respectively. Due to comparable rigidity in both conformations and similar neighboring amide nitrogen distances in the peptide chain, a ^15^N–^15^N PDSD spectrum with 5 s mixing time shows a clear cross-peak for T62′–T63′ with a similar intensity to the T62–T63 cross-peak (Supplementary Fig. 7). In an NHHN spectrum with 200 μs proton–proton mixing time only close amide-proton contacts can be detected, because only distances below ~4 Å give rise to strong cross-peaks^25^. For the K^+^/Rb^+^-favored state, we observed strong cross-peaks for all vicinal amide protons in the sequence _61_LTTVG_65_, which agrees perfectly with the crystal structure (Fig. 3). For the flipped conformation, only the distance between T62 and T63 is ~4.5 Å and indeed lacks the corresponding cross-peak in the ion-free conformational state, whereas all other cross-peaks could still be observed.

### Single SF conformation observed in NaK2K

The different conformational states identified in NaK were not observed in the K^+^ selective mutant NaK2K^13^ (i.e., NaK D66Y and N68D double mutant) prepared under identical conditions. All SF residues could be assigned to a single signal, except T62 which shows an unconfirmed satellite peak (Supplementary Fig. 8). NaK2K is characterized by a SF that is almost indistinguishable from other K^+^ selective channels, such as KcsA, that contain four sequential ion binding sites. The upper part of the SF (Y66-S70), which cannot be detected for NaK, is clearly visible in the spectrum of NaK2K (Supplementary Fig. 8). This indicates an increased rigidity for these residues, consistent with the formation of a canonical K^+^ selective SF.

### K^+^ and Na^+^ permeation by computational electrophysiology

**Fig. 4:**
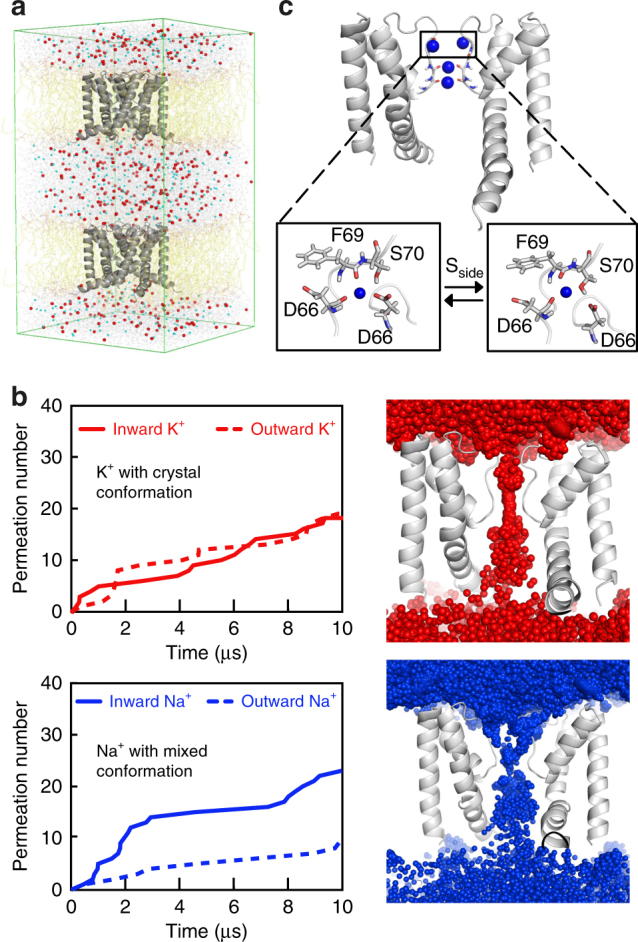
MD simulations of K^+^ and Na^+^ permeation. **a** The simulation system consists of two membranes with NaK (PDB ID: 3E83), surrounded by water and ions, and exhibits a transmembrane voltage gradient (see also Supplementary Fig. 11). **b** Inward and outward permeation events of K^+^ (red) and Na^+^ (blue) vs time. K^+^ simulations were performed with the crystal conformation, while Na^+^ simulations were conducted using a mixture of crystal and T62CO flipped conformations in different subunits. Ion permeation profiles for outward K^+^ and inward Na^+^ are shown on the right. **c** S_side_ coordination of Na^+^ during the inward Na^+^ simulations. The two boxes refer to different coordination states

To check whether the different conformational states stabilized by K^+^ or Na^+^ are functionally relevant to the conduction properties of NaK, we performed atomistic MD simulations with a computational electrophysiology setup previously used to simulate ion permeation in K^+^ selective channels (Fig. 4a)^7,26^. First, we conducted simulations using the crystal structure of NaK (PDB ID: 3E83) with KCl (Methods). Within 10 μs, we observed 18 inward and 19 outward permeation events at a transmembrane voltage of ±460 (±70) mV (Fig. 4b), indicating that the crystal structure is conductive for K^+^.

Next, we employed the same setup to simulate Na^+^ conduction in NaK, however no permeation could be detected within 10 μs. In accordance with previous simulations^27–29^, Na^+^ ions remained tightly bound in the planes formed by backbone carbonyls of T63 or V64 for the duration of the simulations. To determine the conductive conformation of NaK for Na^+^, we performed a series of simulations using combinations of subunits in the flipped and crystal conformations (Supplementary Table 1). Intriguingly, only with mixed conformations were appreciable permeation rates for Na^+^ detected that are comparable to K^+^ conduction; for example, one flipped and three crystal subunits showed 23 inward and 9 outward permeations within 10 μs at a transmembrane voltage of ±560 (±40) mV (Fig. 4b). Although the simulated conductance (0.6 pS for inward K^+^, 0.8 pS for inward Na^+^) is about 44 times lower than observed experimentally (35 pS)^13^, the conductance ratio between the previously simulated MthK channel (MthK: 7.2 pS for outward K^+^)^7^ and NaK from our study is about 9. This agrees relatively well with the experimentally observed single-channel conductance ratio (MthK/NaK: 6.3)^13,30^.

### Asymmetric SF revealed during Na^+^ conduction

**Fig. 5:**
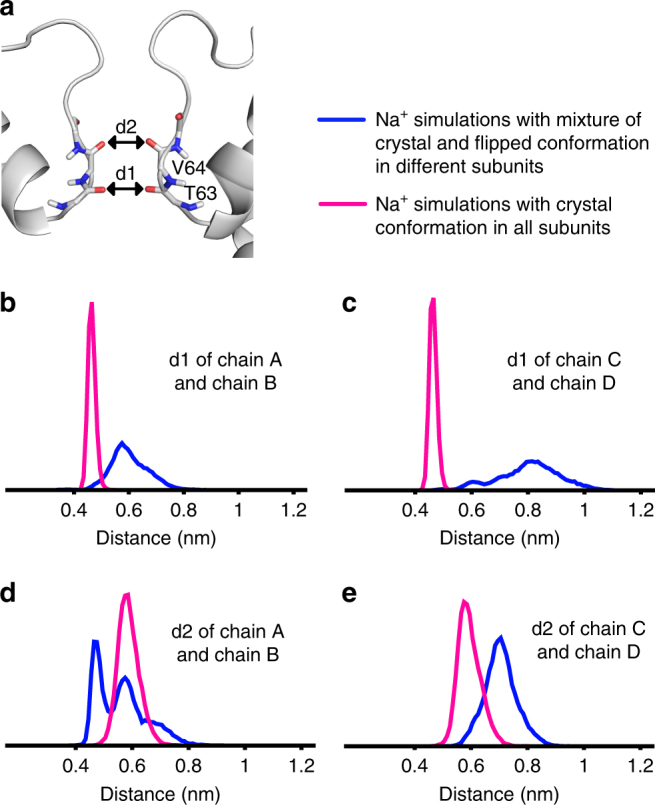
Distribution of the distance between opposing carbonyls from the MD simulations. **a** Distance between two T63 residues is indicated with d1 and between two V64 residues with d2. **b** d1 (T63) of chains A and B, **c** d1 (T63) of chains C and D, **d** d2 (V64) of chains A and B, **e** d2 (V64) of chains C and D. Distances are calculated for the Na^+^ simulations with a mixture of flipped and crystal conformations in different subunits (blue curve, simulation V, Supplementary Table 1), and Na^+^ simulations with the crystal conformation in all subunits (magenta curve, simulation III, Supplementary Table 1)

The SF shows a break in fourfold symmetry by having one or multiple subunits in the T62CO flipped conformation when conducting Na^+^. This leads to an asymmetric deformation of the SF during the simulations, as reflected by the distribution of the distance between opposing backbone carbonyls for residues T63 and V64 (Fig. 5). Ion occupancy (Supplementary Fig. 9) as well as 2D potential of mean force (Supplementary Fig. 10) were calculated from the permeation simulations, which had either a mixture of flipped and crystal conformations or the crystal conformation in all subunits. The comparison suggests that the structural deformation in the SF caused by T62CO flipping significantly reduces the energy barriers for Na^+^ conduction at sites B_23_ and B_34_.

### Side entry ion-conduction pathway for Na^+^ permeation

Surprisingly, we found two distinct ion-conduction pathways for Na^+^ and K^+^ permeation through the SF in the MD simulations. Outward and inward K^+^ currents (see Supplementary Fig. 11 for definition) are dominated by the canonical K^+^ conduction pathway (Supplementary Movies 1 and 2), where multiple K^+^ ions pass through the entire narrow pore of the SF in single file. However, for Na^+^ conduction in both directions, ions use side entries instead of passing through the vestibule and pore entrance (Fig. 4b and Supplementary Movies 3 and 4). Notably, the additional binding site at the side entry (S_side_) (Fig. 4c) is composed of two backbone carbonyls from D66 and F69 and the side chain oxygen from either S70 of the same or D66 of the neighboring subunit. Therefore the side entry pathway is a result of the perpendicular reorientation of the backbone carbonyl from D66 in NaK, which is a tyrosine residue in K^+^ selective channels that points toward the fourfold symmetry axis of the channel.

### Ion occupancy and ion hydration states

During the outward and inward conduction of Na^+^ and K^+^, there were significant differences in the ion occupancies as well as hydration states of ions in the SF. During Na^+^ conduction, only one fully hydrated ion occupied the SF most of the time, while one or more Na^+^ ions bound at S_side_. The ion at B_23_ is weakly populated and short lived (Supplementary Fig. 9). Water molecules were co-transported with the cations during these simulations. In contrast, during K^+^ conduction, usually two dehydrated K^+^ ions simultaneously occupied the S3 and S4 binding sites together with one fully hydrated ion in the vestibule (Supplementary Fig. 12). Additionally, we noticed that a few water molecules were able to be co-transported during the simulations of K^+^ conduction in NaK (Supplementary Movies 1 and 2).

## Discussion

NaK is a prokaryotic non-selective cation channel that is able to conduct both Na^+^ and K^+^ with equally high efficiency. Due to the significant similarity in their SF sequences as well as their non-selective nature, NaK has been considered a bacterial homolog of eukaryotic cyclic-nucleotide-gated channels. In the present study, a combined use of ssNMR and MD simulations revealed two different conformational states of the SF that exist simultaneously in NaK and are stabilized by different ions. The K^+^/Rb^+^-favored state was confirmed as the crystal conformation, while the ion-free state corresponds to the T62CO flipped conformation. Most intriguingly, Na^+^ favors the ion-free state and results in an asymmetric deformation of the SF.

The structural complexity of Na^+^ binding was experimentally supported by the appearance of additional peaks in ssNMR spectra of NaK at high Na^+^ concentration (150 mM NaCl, Supplementary Fig. 6), which were not visible with K^+^ at the same concentration. This indicates an importance of conformational dynamics during Na^+^ conduction, since the additional peaks appear only under high Na^+^ conditions.

We compared the distance information of the ion-free and K^+^/Rb^+^-favored states (obtained from the ssNMR spectra) with the SF conformations seen in KcsA. The conductive and collapsed conformations of KcsA show no significant difference in the distances between neighboring amide protons (Supplementary Fig. 13). Thus, the conformational dynamics observed in the SF of NaK are absent in the K^+^ selective channel KcsA. However, comparison of the dipolar-coupling and scalar-coupling based NMR experiments suggests that the dynamics of the C- and N-terminal regions are relatively slow in NaK. This behavior is most likely important for channel gating, which is in accordance with the activation gating mechanism of KcsA by allosteric transmembrane coupling^31,32^.

To further validate the functional relevance of the conformational plasticity of the SF in NaK, we performed the ssNMR experiments also on a K^+^ selective NaK double mutant (NaK2K) under otherwise identical conditions. Here we observed only a single conformational state of the SF, which is in strong contrast to NaK. Furthermore, the upper part of the SF (Y66-S70) became visible, as would be expected from a KcsA-like SF conformation. These results eliminate the possibility that the observed conformational heterogeneity in NaK is a result of our sample preparation.

Computational electrophysiology enabled the determination of the conductive conformation for Na^+^, while the crystal structure is only conductive for K^+^. Efficient Na^+^ conduction in NaK requires conformational plasticity and the resulting asymmetric deformation of the SF.

**Fig. 6:**
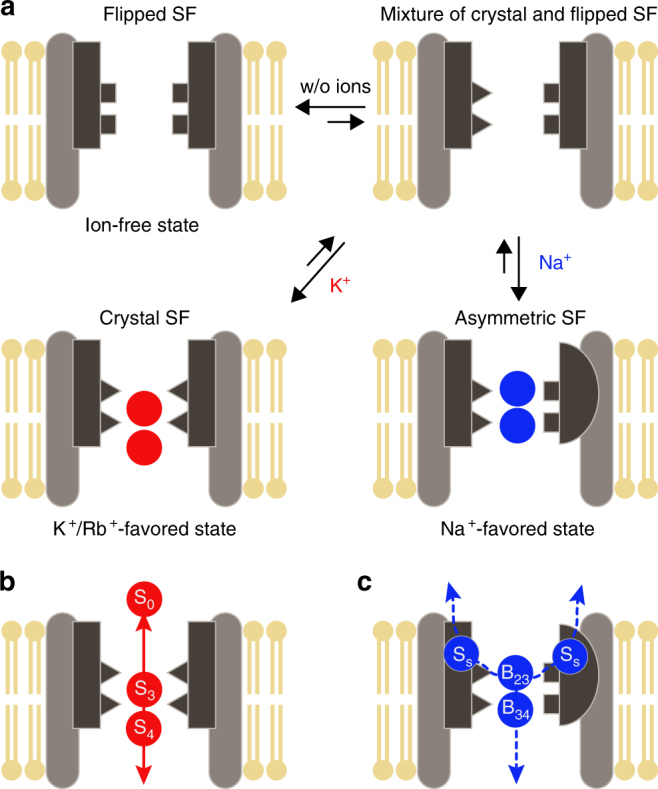
Schematic model of the non-selective ion-conduction mechanism in NaK. **a** Equilibrium of NaK conformations without ions or in the presence of Na^+^ or K^+^. Plasticity in the SF enables Na^+^ binding, further resulting in an asymmetric deformation of the SF. **b** K^+^ conduction pathway from the MD simulations. **c** Na^+^ conduction in both directions uses the four side entries (dashed line). The additional binding sites (S_side_) at the side entry are labeled as S_S_

Most importantly, we show that the permeation mechanisms of K^+^ and Na^+^ are remarkably different. In NaK several K^+^ ions move in a single file along the axis of the SF via a loosely coupled direct knock-on mechanism. Although a few water molecules could be co-transported during K^+^ conduction, the permeation mechanism is similar to that proposed for K^+^ selective channels using the same computational approach^7^. Intriguingly, for the inward and outward Na^+^ current, we identified a different pathway in which Na^+^ uses a peculiar side entry into the SF (Fig. 6c). The additional binding site at the side entry is attributed to the perpendicular reorientation of the backbone carbonyl of D66 in NaK. We also observed the side entry pathway for K^+^ permeations in a set of simulations, where V64CO flipping resulted in a similar kind of asymmetric deformation of the SF. Nevertheless, since the V64CO flipped conformation in the presence of K^+^ was not supported by the ssNMR data, we do not consider this side entry pathway to be physiologically relevant for K^+^.

Moreover, in marked contrast to the K^+^ conductive state, the lower part of the SF in the Na^+^ conductive state becomes wider (Fig. 5). The wider pore enables only one fully hydrated Na^+^ ion to reside in the SF with a short occupation time at B_23_. This result further indicates that during Na^+^ permeation ions do not need to be fully dehydrated. The Na^+^ permeation mechanism in NaK resembles the one that was observed for Na^+^ conduction through the open conformation of a bacterial voltage-gated Na^+^ channel^33^. As the SF of the NavM channel^34,35^ is significantly wider than that of K^+^ selective channels, the ions are hydrated at all sites during the Na^+^ permeation simulations. Previous studies suggested that dehydration of the smaller Na^+^ ions requires much more energy than of larger K^+^ ions. Conduction through the SF without full dehydration is thus beneficial for Na^+^ ions^36,37^. Furthermore, it should be noted that the SF of the less selective Na^+^ channels, as well as of several other recently structurally elucidated  non-selective ion channels^38–40^, is considerably wider than the SF of highly K^+^ selective channels. Consequently, we propose that the structural plasticity and the ability of adapting the size of the SF in NaK are key molecular determinants for its ion non-selectivity. Previous NMR and crystallographic studies on both NaK and KcsA did not reveal the same degree of conformational plasticity in the SF^15,17^. We believe our model complements the previously proposed thermodynamic and kinetic binding mechanisms^10^ as well as the single-barrier mechanism of non-selectivity in NaK^11,13^ and thereby provides a more thorough understanding of ion selectivity in K^+^ selective and non-selective channels.

## Methods

### Sample preparation

The *NaK* gene without the first 17 amino acids was cloned into a pET28a expression vector (Supplementary Table 2) with an N-terminal hexahistidine (His_6_) sequence. To prepare the [^13^C, ^15^N]-labeled samples, the protein was expressed in BL21(DE3)-Gold cells (Agilent) in M9 medium with ^13^C-glucose as the sole carbon source and ^15^NH_4_Cl as the sole nitrogen source. The cultures were grown for 12 h at 20 °C after induction. The membrane fraction was isolated and solubilized in 20 mM DM (*n*-decyl-beta-d-maltopyranoside). The protein samples were purified by Ni-NTA affinity chromatography and size exclusion chromatography either without cations or in the presence of 50 or 150 mM Na^+^, 50 or 150 mM K^+^, or 50 mM Rb^+^. The concentration of purified NaK was determined by the A280 method with the calculated extinction coefficient. The NaK tetramers were reconstituted into asolection liposomes at a lipid/tetramer ratio of 40:1 using dialysis. Proteoliposomes were collected by ultracentrifugation at 300,000×*g* for two hours at 4 °C.

### Single-channel conductance measurements

After dialysis, the protein/lipid mixture was extruded 20 times through a 100 nm polycarbonate membrane filter (Whatman, Newton, MA) with an Avanti mini-extruder to make unilamellar liposome vesicles. Channel conductance measurements in planar lipid bilayers were conducted on an Ionovation Compact device (Osnabrück, Germany). Two polycarbonate compartments with a volume of 1.2 mL were separated by a sheet of TEFLON foil with 25 μm thickness and 50−100 μm aperture diameter. The artificial lipid bilayer was formed in the aperture through the method of painting. The cis-chamber was filled with 15 mM NaCl, 150 mM KCl, 5 mM Mops, 5 mM Tris-HCl, pH 7.0, and the trans-chamber with 150 mM NaCl, 15 mM KCl, 5 mM Mops, 5 mM Tris-HCl, pH 7.0. Voltage was applied across the bilayer using Ag/AgCl electrodes immersed in each chamber. With the grounded cis-compartment, a positive potential indicating a higher potential in the trans-chamber was applied. Planar lipid bilayer formation was monitored optically or by capacitance measurements. After successful formation of a stable bilayer in the aperture, NaK proteoliposomes were added to the trans-chamber next to the bilayer. Fusion of the protein and the planar lipid bilayer was detected through observation of channel conductance. Signal acquisition and analysis were performed using the pCLAMP software (Axon Instruments). Single-channel conductance events were identified automatically and analyzed using the Clampfit 10.0 software (Axon Instruments). Representative traces at bi-ionic conditions demonstrated that the NaK channel could open at both positive and negative voltages with a reversal potential near zero, indicating that NaK could conduct both Na^+^ and K^+^ ions with low ion selectivity (Supplementary Fig. 1).

### ssNMR spectroscopy and analysis

The proteoliposome pellets were transferred into 3.2 mm magic-angle spinning (MAS) rotors and used for ssNMR analysis. SsNMR spectra required for chemical shift assignment were recorded on a 16.4 T wide-bore NMR spectrometer (700 MHz, Bruker BioSpin) equipped with a 3.2 mm triple-resonance Efree MAS probe. Spectra were recorded at 17 kHz MAS rate and calibrated with external DSS (4,4-dimethyl-4-silapentane-1-sulfonic sodium salt) as a proton chemical shift reference. Intra-residue spin system correlations were obtained from 3D NCACB and band-selective homonuclear cross-polarization (BSH-CP)^41,42^ based NCACO spectra, whereas inter-residue connections were provided by 3D CANCO and BSH-CP based NCOCA and NCOCACB spectra. As an illustration, a sequential walk from A53 to S57 is shown in Supplementary Fig. 2. The inter-residue Cα_i_-Cα_i-1_ connections were further confirmed by a 2D CANCOCA spectrum. Subsequently, the side chain carbon chemical shifts were assigned with a 3D NCACX spectrum. A ^13^C-^13^C proton-driven spin diffusion (PDSD)^43^ spectrum was recorded at 11 kHz MAS with 20 ms mixing time to assist the side chain assignment (Supplementary Fig. 3). An INEPT-based ^1^H–^13^C correlation spectrum was recorded at 11 kHz MAS to detect flexible residues. The complete chemical shift list for NaK in the presence of 50 mM Na^+^ is provided in Supplementary Table 3.

For the detection of short (<4 Å) distances between amide protons in the SF of NaK, an NHHN spectrum was recorded at 16.4 T and 11 kHz MAS using 200 μs proton–proton mixing time. As a reference, a 2D ^15^N–^15^N PDSD spectrum was recorded at 11 kHz MAS with 5 s mixing time.

The NCA and NCO spectra that were used to detect cation-specific chemical shift changes in NaK were recorded on an 18.8 T wide-bore NMR spectrometer (800 MHz, Bruker BioSpin) equipped with a 3.2 mm triple-resonance Efree MAS probe at 11 kHz MAS rate. The resulting spectra are shown in main text Fig. 2 (excerpts) and Supplementary Fig. 6 (full spectra). The chemical shifts of the SF residues under different ionic conditions (<1 μM; 50 mM Na^+^; 50 mM K^+^; 50 mM Rb^+^) are listed in Supplementary Table 4.

During all experiments, the sample temperature was kept constant at 4–8 °C, as measured by the temperature-dependent position of the water resonance^44^. High-power proton decoupling using the sequence SPINAL-64^45^ with a radio frequency amplitude of about 83 kHz was applied during evolution and detection periods.

### Molecular dynamics simulations

In all simulations, the NaK channel was embedded in a hydrated 1-palmitoyl 2-oleoyl-phosphatidylcholine (POPC) lipid bilayer with all atoms, including those in the lipids and water molecules, represented explicitly^46^.

The starting structure in the simulations was based on the high-resolution crystal structure of NaK (PDB ID: 3E83). All titratable residues were left in the dominant protonation state at pH 7.0. For the computational electrophysiology simulations, the F92A mutant instead of the wild-type (WT) channel was employed, as previously performed electrophysiology experiments revealed a significant increase in ion-conduction rates for the F92A mutant as compared to the WT channel^47^.

MD simulations were carried out using GROMACS 5.0^48,49^. We used the AMBER99sb force field^50^ and the SPC/E water model^51^ for the equilibrium and production simulations of the proteins. Parameters for ions and lipids were derived from references^52,53^. Short-range electrostatic interactions were calculated with a cutoff of 1.0 nm, whereas long-range electrostatic interactions were treated by the particle mesh Ewald method^54,55^. The cutoff for van-der-Waals interactions was set to 1.0 nm. Virtual sites were used for protein hydrogen atoms during the simulations, which allows for a 4 fs time step for integration. The simulations were performed at 300 K with a velocity rescaling thermostat^56^. The pressure was kept constant at 1 bar by means of a semi-isotropic Parrinello–Rahman barostat^57,58^. All bonds were constrained with the LINCS algorithm^59^.

To equilibrate the system, a short 20 ns simulation was performed by position-restraining all heavy atoms of the NaK channel with a force constant of 1000 kJ mol^−1^ nm^−2^ to the starting structure. The system was further equilibrated for 20 ns by releasing the position restraints.

SsNMR spectra revealed two well-separated signals for SF residues V64 and G65, suggesting that the two corresponding conformations of the SF are in very slow exchange on the NMR timescale. This result further indicates that interconversion from one conformation to the other is not expected to take place within ns–μs MD simulations. As flipping of backbone carbonyls has been observed in some structures of mutants and in simulations of KcsA^6,23,24^, we assumed that the second signal set of the ssNMR spectra corresponds to a backbone carbonyl flipped conformer. In order to investigate which carbonyl flipped conformers are thermodynamically stable, we first generated a set of carbonyl flipped conformers by manually flipping the backbone carbonyls of T62, T63, V64, and G65 in the SF by ~180° using the program Coot^60^. Two other residues in the SF (D66 and G67) were not tested, as no NMR signals were observed for these residues, indicating a fast conformational exchange on the ns–μs timescale. The manually flipped conformers were equilibrated first by position-restraining all heavy atoms of the SF with a force constant of 1000 kJ mol^−1^ nm^−2^ to the starting structure. Starting from these equilibrated structures, we further tested the stability of the carbonyl flipped conformers by performing 5 independent runs of 20 ns MD simulations without position restraints. Among all carbonyl flipped conformers, only the T62CO flipped conformer was stable during these simulations, while for the other conformers the backbone carbonyl quickly returned to the crystal conformation.

For the computational electrophysiology simulations^61^, the equilibrated system was duplicated along the *z* direction and transmembrane potential gradients were generated by introducing two more K^+^ or Na^+^ ions in compartment (a) than in compartment (b) (Supplementary Fig. 11). During the MD simulations, the number of ions in these two compartments was kept constant by an additional algorithm^61^. The charge difference leads to a positive voltage difference for the upper channel and a negative voltage difference for the lower channel (Supplementary Fig. 11). The resulting membrane potential can be calculated by double integration of the charge distribution using the Poisson equation as implemented in the GROMACS tool g_potential^62^. During the simulations an outward permeation event was counted when an ion moved from the cavity to the SF and another ion left the SF, while an inward permeation event was counted when an ion moved from the extracellular side to the SF and another ion left the SF. Snapshots of the NaK simulations including ions and water molecules are shown in Supplementary Figure 12.

### Data availability

Data supporting the findings of this manuscript are available from the corresponding authors upon reasonable request. The NaK chemical shifts have been deposited in the Biological Magnetic Resonance Data Bank under accession codes 27219 (the ion-free conformation) and 27220 (the K^+^/Rb^+^-favored conformation).

## Electronic supplementary material

Supplementary Information

Description of Additional Supplementary Files

Supplementary Movie 1

Supplementary Movie 2

Supplementary Movie3

Supplementary Movie 4
