# Supplementary material for: A single NaK channel conformation is not enough for non-selective ion conduction
Source: Nat Commun. 2018 Feb 19;9:717. doi: 10.1038/s41467-018-03179-y (PMC5818664; doi:10.1038/s41467-018-03179-y)
Supplement: Supplementary file 1 — Supplementary Information [file 41467_2018_3179_MOESM1_ESM.pdf]

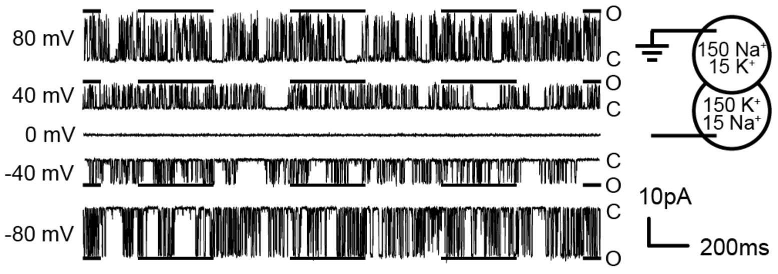

Supplementary Figure 1 | Single-channel conductance of NaK after reconstitution into lipid bilayers at various membrane potentials. Currents were recorded with 15 mM NaCl, 150 mM KCl, 5 mM Mops, 5 mM Tris-HCl, pH 7.0 in the cis-chamber and 150 mM NaCl, 15 mM KCl, 5 mM Mops, 5 mM Tris-HCl, pH 7.0 in the trans-chamber. Solid line: closed (C); dashed line: open (O).

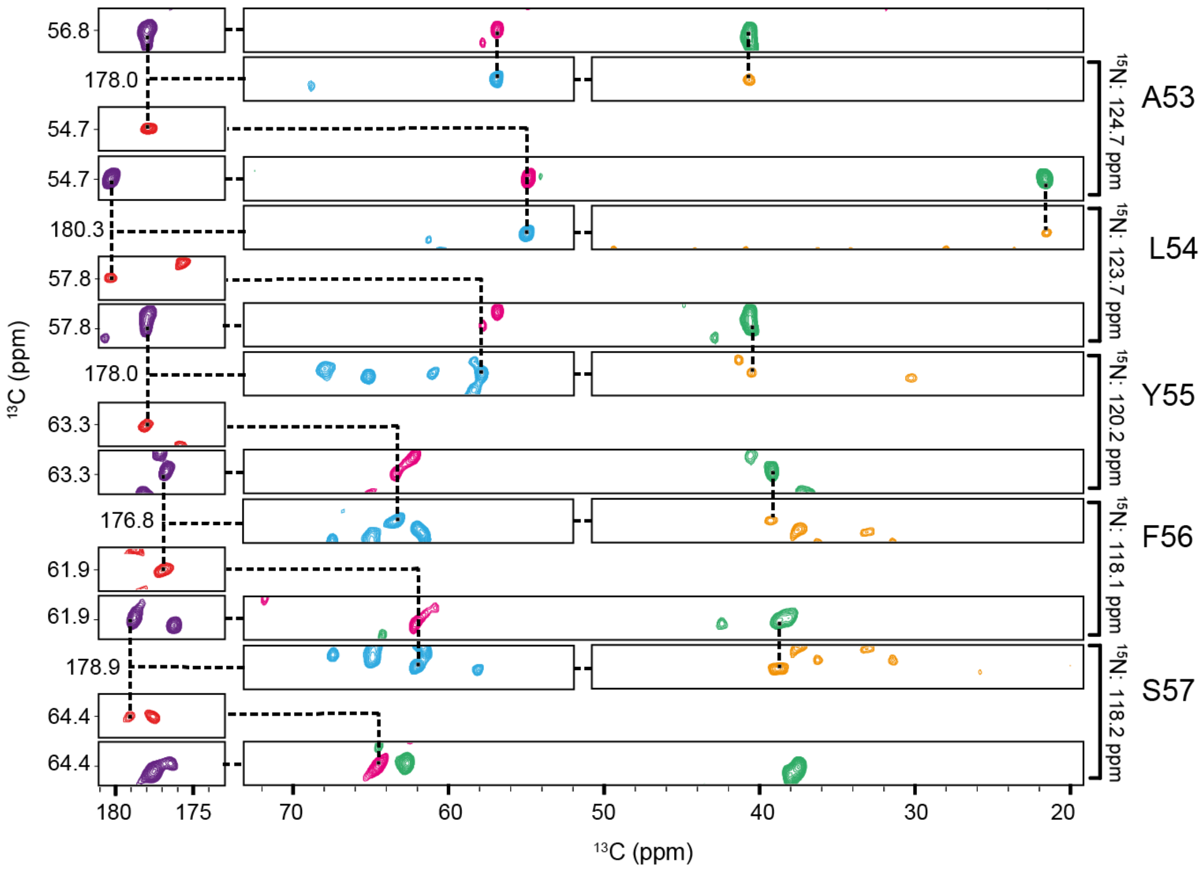

Supplementary Figure 2 | Sequential 'backbone walk' using the recorded 3D spectra for A53 to S57. Contours from the NCACO spectrum (purple), the NCACB spectrum with positive CA signals (magenta) and negative CB signals (green), the NCOCA spectrum (light blue), the NCOCACB spectrum with negative CB signals only (orange), and the CONCA spectrum (red) are shown. Dashed lines connect signals in different spectra that exhibit the same chemical shift. All spectra were recorded on a sample of uniformly [^{13}C,^{15}N]-labeled NaK in the presence of 50 mM Na^+. The experiments were conducted on a 16.4 T wide-bore NMR spectrometer at 17 kHz MAS rate.

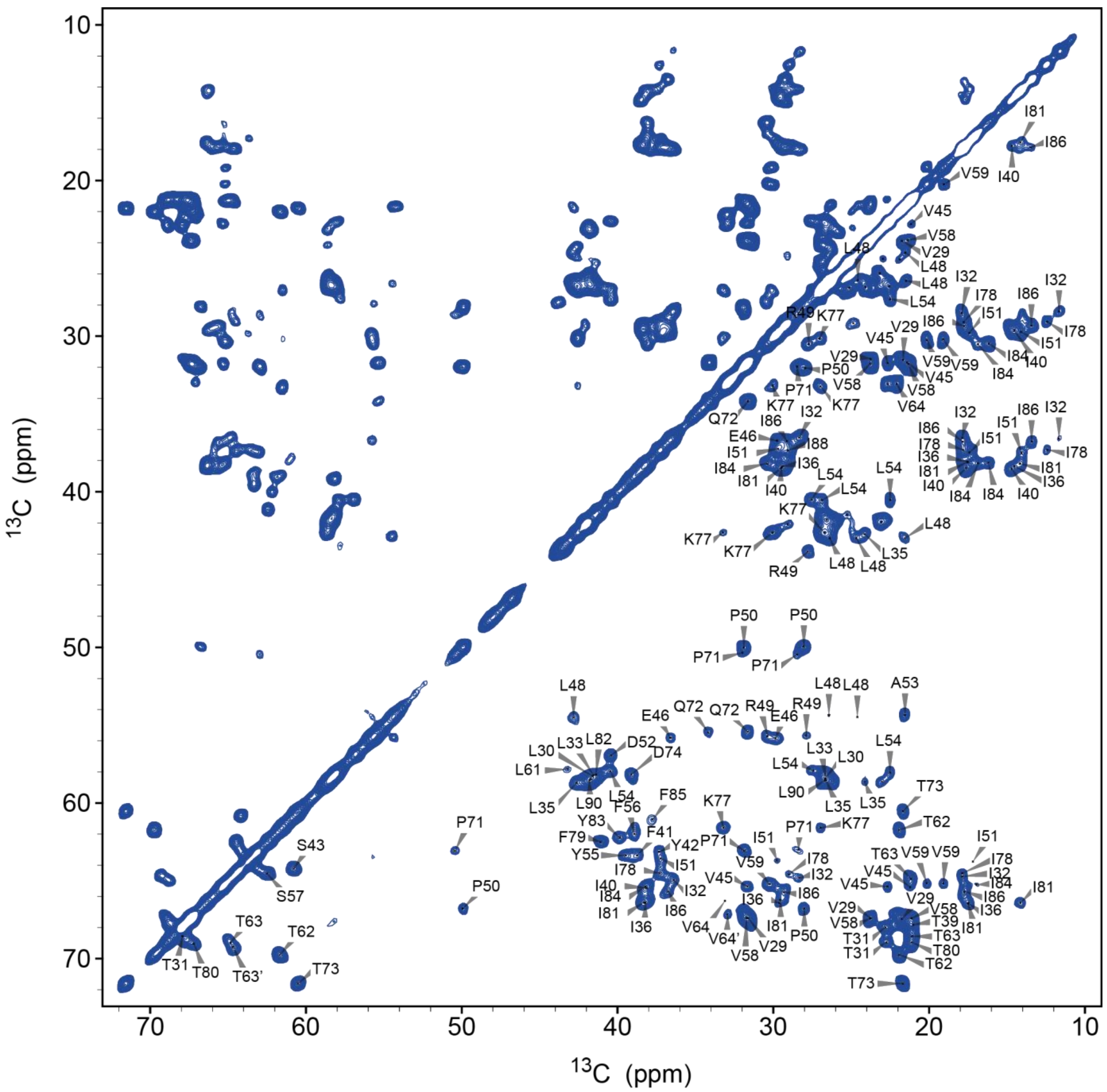

Supplementary Figure 3 | 2D ^{13}C-^{13}C PDSD correlation spectrum of uniformly [^{13}C,^{15}N]-labeled NaK in the presence of 50 mM Na^{+}. The spectrum was recorded on a 16.4 T wide-bore NMR spectrometer at 11 kHz MAS rate. The ^{13}C-^{13}C mixing time was set to 20 ms.

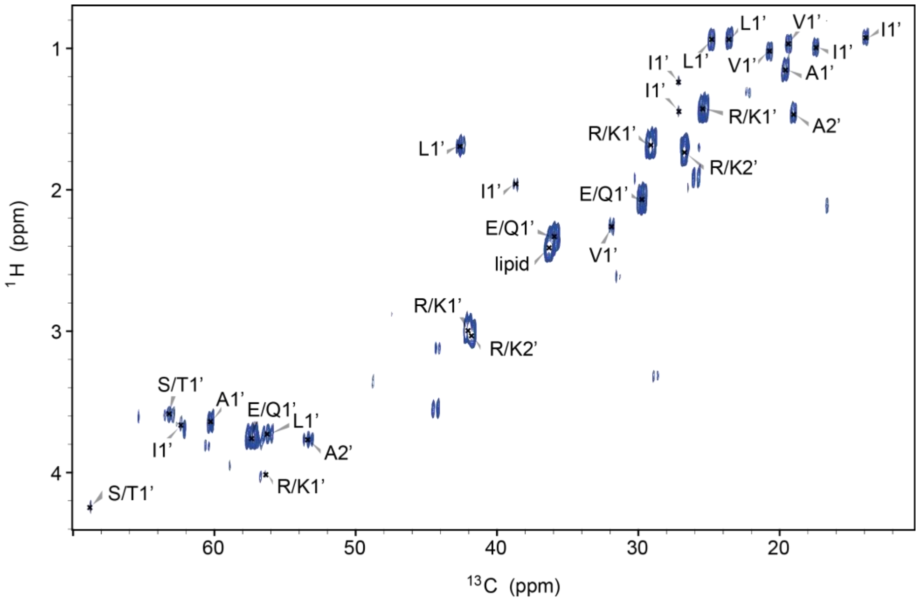

Supplementary Figure 4 | Scalar based HC spectrum of uniformly [^{13}C, ^{15}N]-labeled NaK in the presence of 50 mM Na$^+$. The observed spin systems correlate well to the C-terminal residue types ($_{105}$PSILSNRKKE$_{114}$) following the M2 helix and the N-terminal alanine residue.

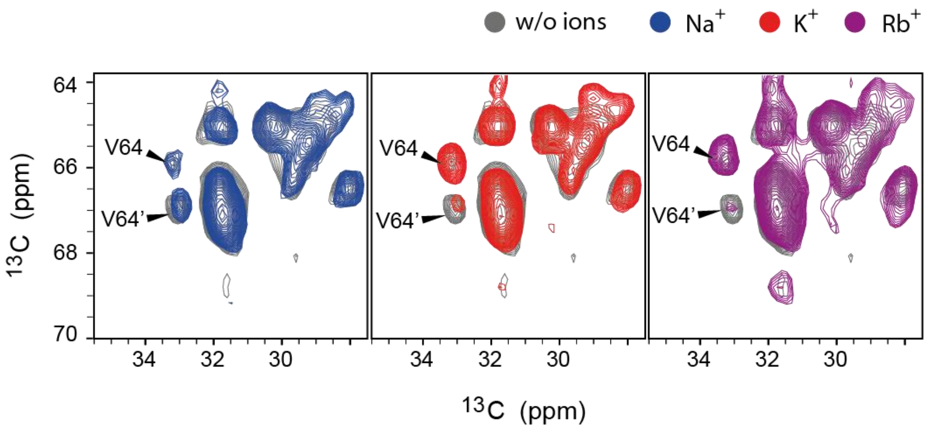

Supplementary Figure 5 | Excerpts of the V64 cross-peaks from PDSD spectra. Close-up of the CA-CB cross-peaks of V64. Spectra are presented in grey for sample A (< 1 µM ions), in blue for sample B (with 50 mM Na^+), in red for sample C (with 50 mM K^+), and in purple for sample D (with 50 mM Rb^+).

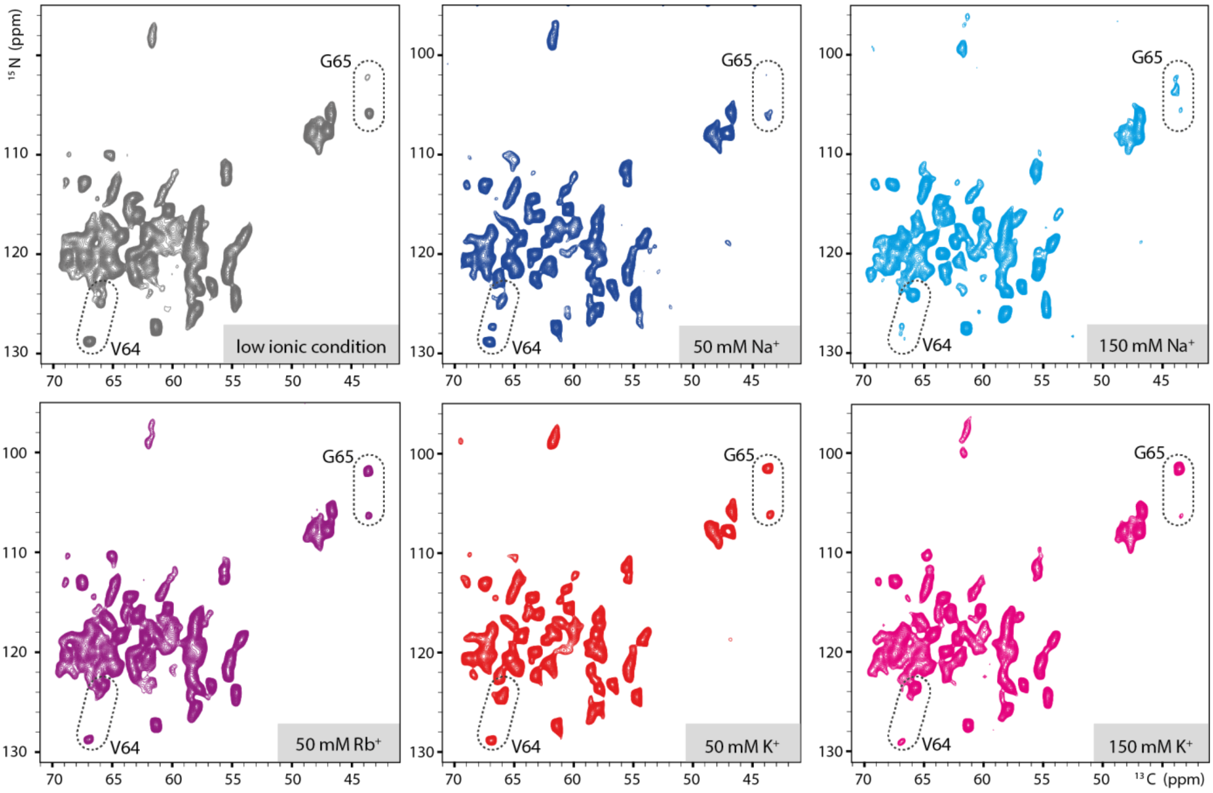

Supplementary Figure 6 | Comparison of 2D NCA spectra for NaK under different ionic conditions. The spectra show NaK at low ionic (< 1 µM) conditions (grey contours), and in the presence of 50 mM Na^+ (blue), 150 mM Na^+ (cyan), 50 mM Rb^+ (purple), 50 mM K^+ (red), and 150 mM K^+ (magenta).

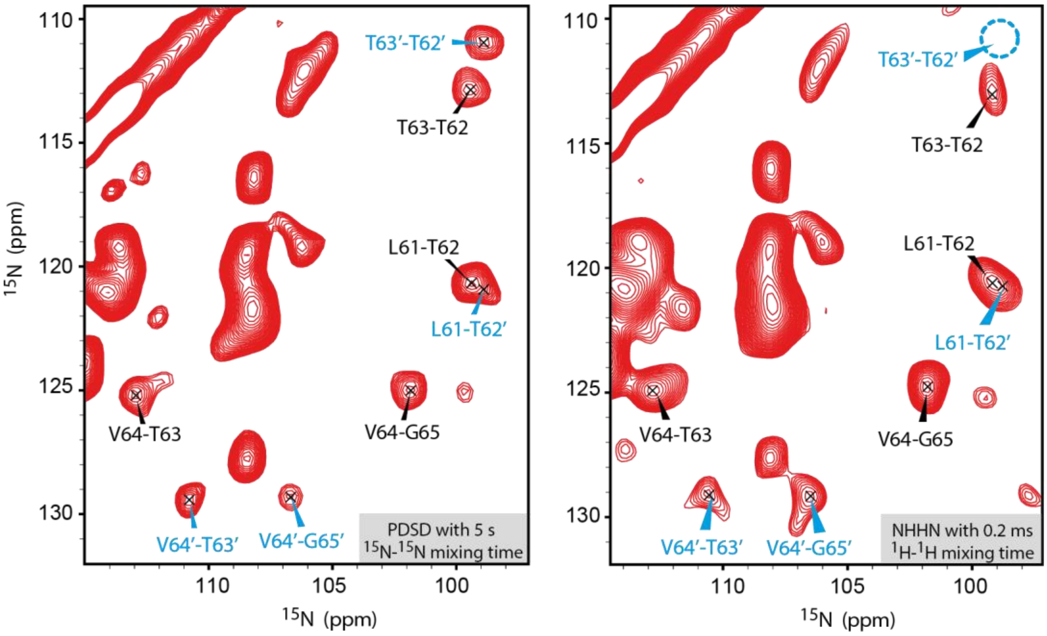

Supplementary Figure 7 | Comparison of ^{15}N-^{15}N PDSD spectrum and NHHN spectrum. The ^{15}N-^{15}N PDSD spectrum shows a clear T62’-T63’ cross-peak, whilst the NHHN spectrum does not. The spectra were recorded on a 16.4 T wide-bore NMR spectrometer at 11 kHz MAS rate.

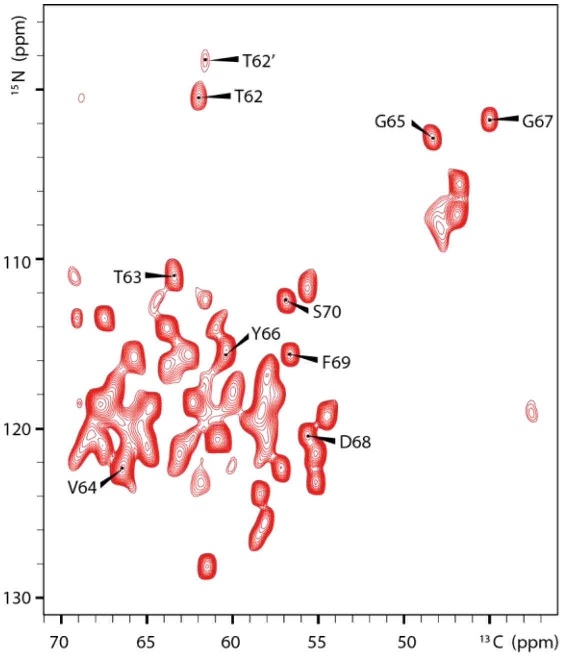

Supplementary Figure 8 | 2D ^{15}N-^{13}CA correlation spectrum of uniformly [^{13}C,^{15}N]-labeled NaK2K in the presence of 50 mM K^{+}. The spectrum shows a single set of peaks for the SF (assignment indicated; T62' is not confirmed by sequential assignment). The spectrum was recorded on a 16.4 T wide-bore NMR spectrometer at 17 kHz MAS rate.

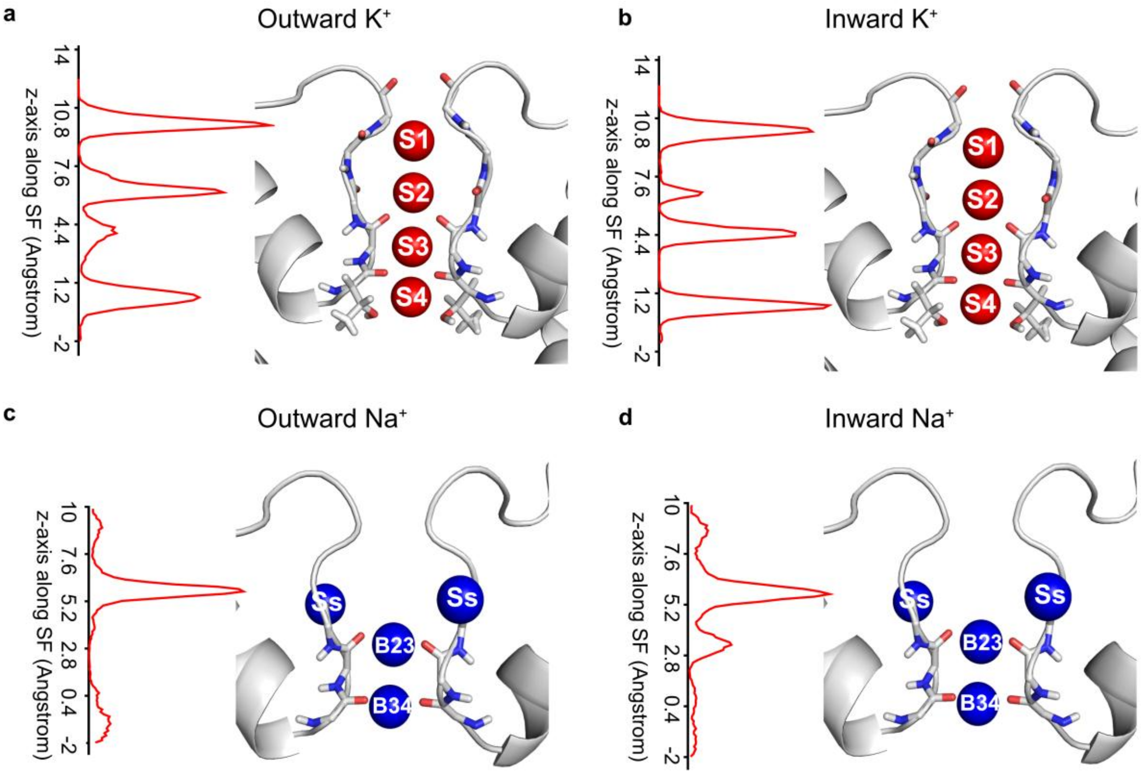

Supplementary Figure 9 | Ion occupancy along the SF axis during simulations. Ion occupancy during (a) K^+ outward and (b) K^+ inward, as well as (c) Na^+ outward and (d) Na^+ inward simulations. Details of outward and inward K^+ simulations are listed as simulation I in Supplementary Table 1, while outward and inward Na^+ simulations are listed as simulation V in Supplementary Table 1.

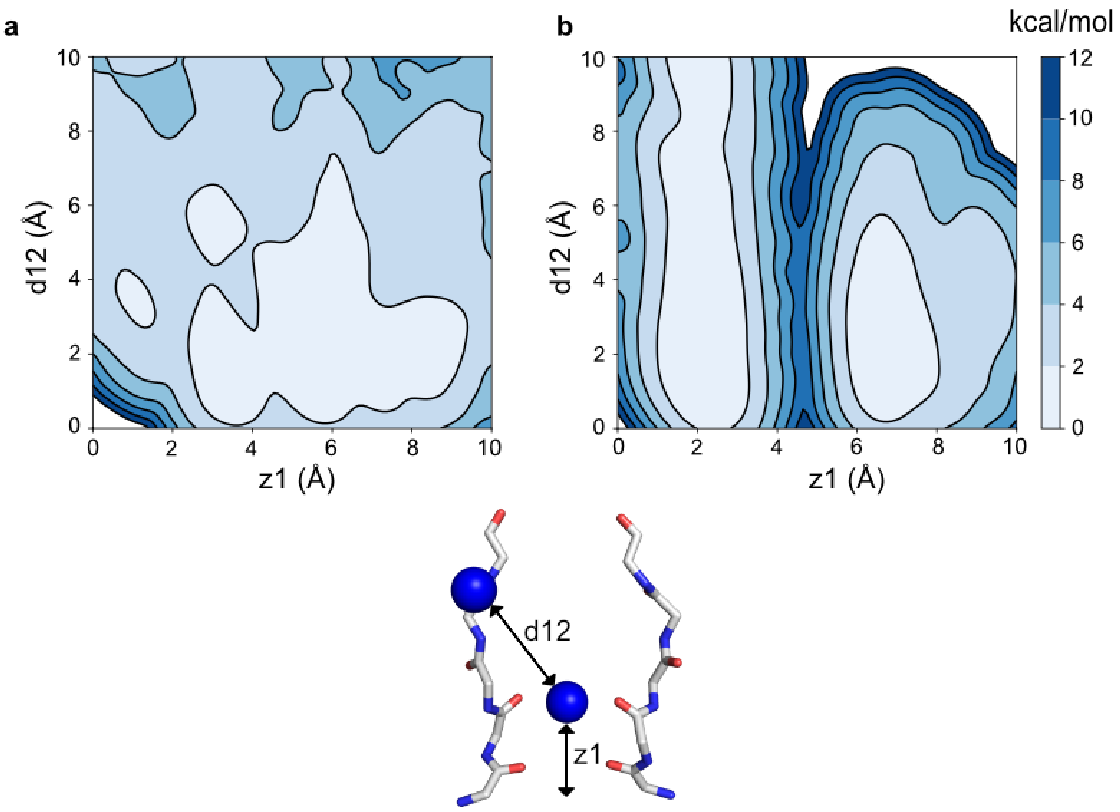

Supplementary Figure 10 | Comparison of potential of mean force of sodium permeation simulations. (a) Na^+ simulations with a mixture of flipped and crystal conformations in different subunits (simulation V, Supplementary Table 1). (b) Na^+ simulations with the crystal conformation in all subunits (simulation III, Supplementary Table 1). Here Na^+ was tightly bound to the SF with little ion movement due to high free energy barriers > 8 kcal/mol. The reaction coordinate d12 corresponds to the distance between the Na^+ in the SF and the Na^+ at the side entrance, and z1 corresponds to the position of the Na^+ in the SF relative to the lowest position of T63.

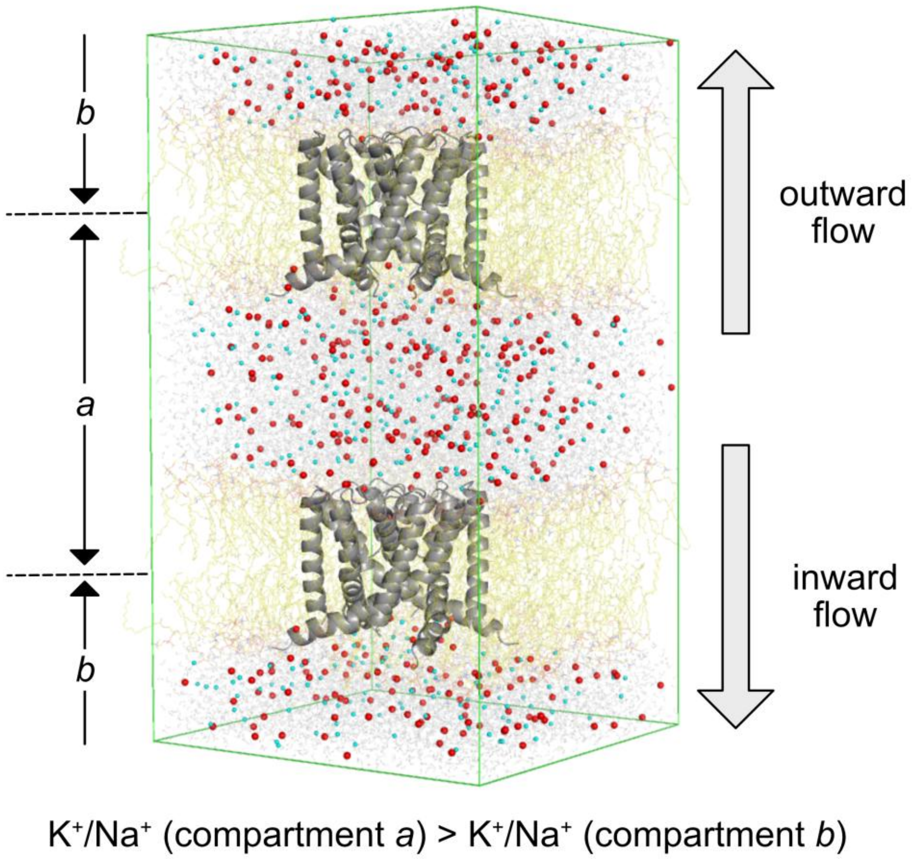

Supplementary Figure 11 | The system used in the computational electrophysiology simulations, consisting of two membrane layers (lipids in yellow). Each layer includes one NaK channel (gray cartoon: PDB ID: 3E83), surrounded by water, K^+ ions (red balls) and Cl^- ions (cyan balls). Periodic boundary conditions create two compartments (*a* and *b*) with two more K^+/Na^+ ions in *a* than in *b*. Thus, a positive transmembrane voltage gradient is established across the upper channel, while a negative voltage gradient is established across the lower channel.

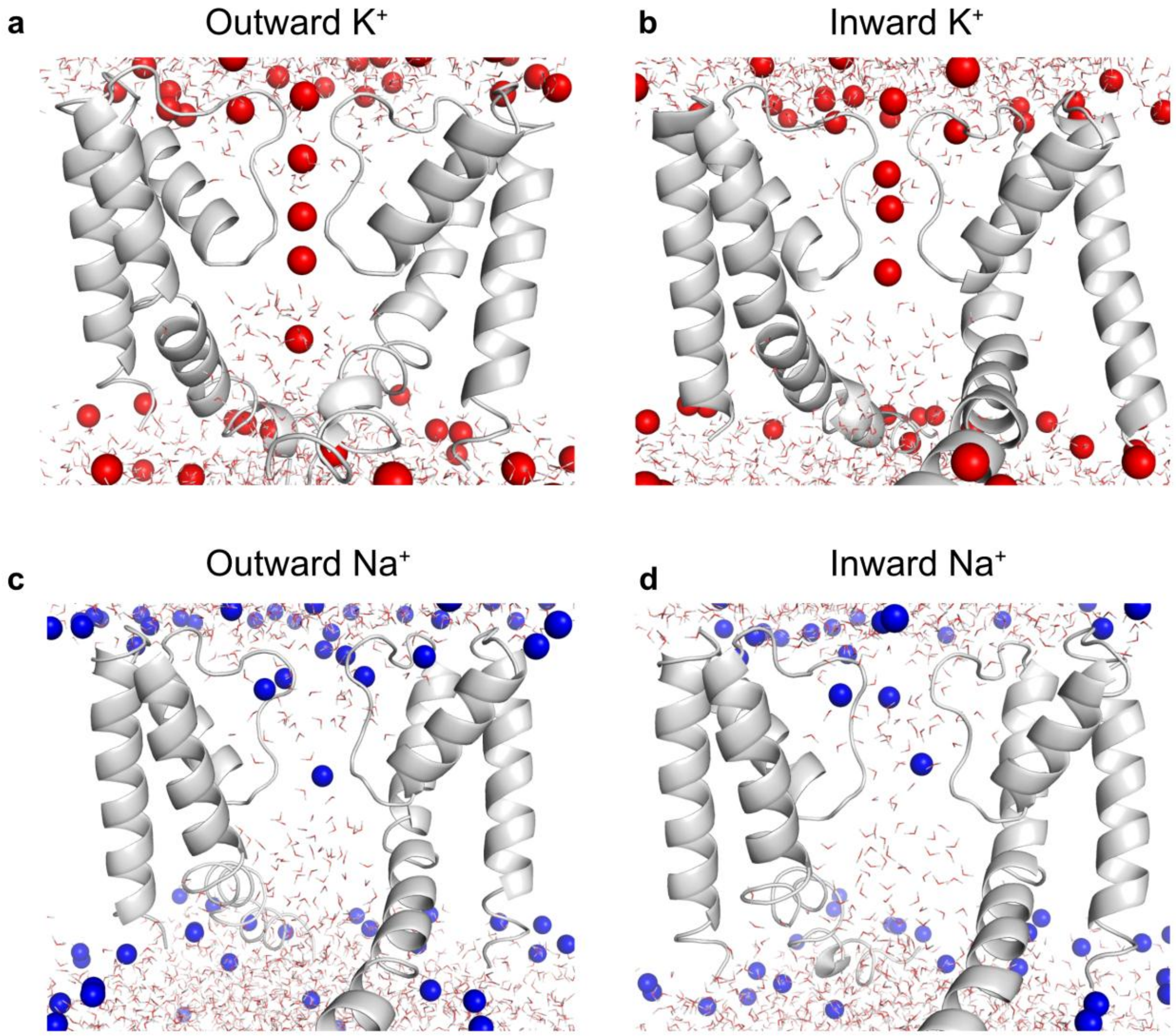

Supplementary Figure 12 | Snapshots of the NaK simulations. (a) Outward K^+ conduction (simulation I, Supplementary Table 1), (b) inward K^+ conduction (simulation I, Supplementary Table 1), (c) outward Na^+ conduction (simulation V, Supplementary Table 1), and (d) inward Na^+ conduction (simulation V, Supplementary Table 1) reveal differences in the hydration states of K^+ and Na^+ ions in the SF during permeation.

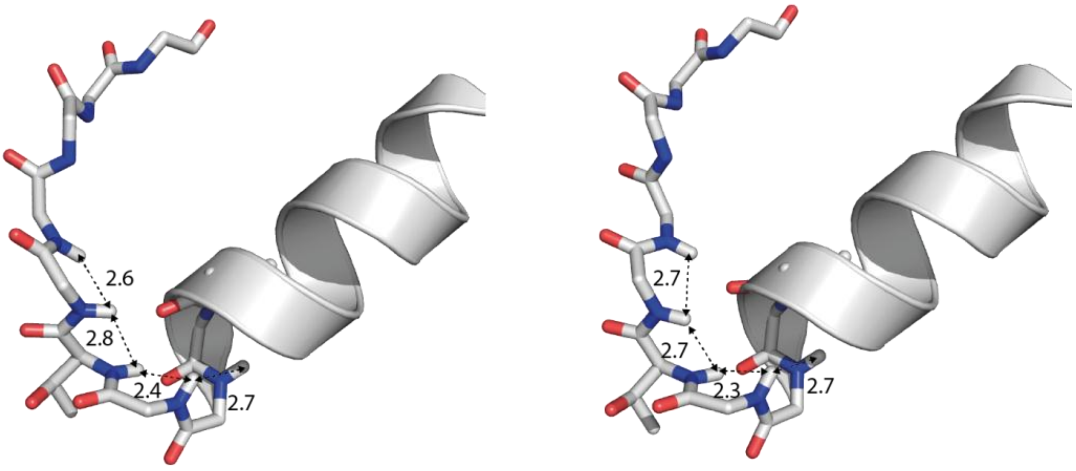

KcsA in low concentration K^+ KcsA in high concentration K^+

Supplementary Figure 13 | The structure of the SF from KcsA in low and high concentration K^+ buffers, known as the collapsed and conductive SF, respectively.

Supplementary Table 1 | Simulation details for computational electrophysiology simulations: production simulations varied in ion type, SF conformations and SF starting pattern.

**Simulation set**	**I**	**II**	**III**	**IV**	**V**	**VI**	**VII**
**Ion type**	K^+	Na^+	Na^+	Na^+	Na^+	Na^+	Na^+
**SF conformations**	4 crystal	4 crystal	4 crystal	3 crystal + 1 flipped	3 crystal + 1 flipped	1 crystal + 3 flipped	4 flipped
**Number of lipids**	424 POPC	424 POPC	424 POPC	424 POPC	424 POPC	424 POPC	424 POPC
**Number of water molecules**	22510	22510	22510	22510	22510	21592	21592
**Number of ions**	600 mM 440 K^+ 424 Cl^-	600 mM 440 Na^+ 424 Cl^-	600 mM 440 Na^+ 424 Cl^-	600 mM 440 Na^+ 424 Cl^-	600 mM 440 Na^+ 424 Cl^-	600 mM 440 Na^+ 424 Cl^-	600 mM 440 Na^+ 424 Cl^-
**SF starting pattern (S_1-S_4)**	KKKK	NaNaNaNa	wwww	NaNaNaNa	wwww	wwww	wwww
**Independent simulations**	10	10	10	10	10	10	10
**Total simulation time (µs)**	10	10	10	7	10	10	10
**Total inward permeations**	18	0	0	6	23	18	0
**Total outward permeations**	19	0	0	0	9	6	0
**Voltage (mV)**	460±70	470±30	440±40	430±80	560±40	520±50	410±50

Supplementary Table 2 | Primer sequences.

p28-NaK_forward	GGGAATTCCATATGGCGTGGAAAGATAAAG
p28-NaK_reverse	CCGCTCGAGCTACTCTTTTTTTCTATTCG

Supplementary Table 3 | Chemical shift assignments for NaK in the presence of 50 mM Na^{+}.

	N	CO	Cα	Cβ	Cγ	Cδ	Other
**V29**	118.4	178.3	67.37	31.49	23.82/21.72		
**L30**	118.4	180.1	58.17	41.67	26.63		
**T31**	120.8	176.2	68.49	67.94			
**I32**	121.4	178.4	64.77	36.49	28.40/17.88	11.63	
**L33**	119.1	180.2	58.23	41.68	26.69		
**T34**	121.1	178.4	68.53				
**L35**	124.9	180.5	58.50	42.62	26.54	24.15	
**I36**	121.9	177.3	66.29	38.26	29.55/17.52	14.53	
**S37**	116.6	176.4	63.92	62.98			
**G38**	108.0	174.9	47.16				
**T39**	118.7	177.6	67.92	68.04	21.03		
**I40**	120.5	178.3	65.37	38.39	29.52/17.65	14.67	
**F41**	122.6	177.3	63.23	38.56			
**Y42**	116.3	178.3	62.93	37.56			
**S43**	113.6	175.4	60.65	64.19			
**V45**	121.8	177.2	65.26	31.68	22.69/21.24		
**E46**	111.6	177.0	55.62	29.73	36.68	181.1	
**G47**	105.7	174.2	46.70				
**L48**	119.0	177.9	54.17	42.82	26.41	24.64/21.57	
**R49**	122.0	176.5	55.45	30.38	27.83	43.82	Cζ:160.0
**P50**	136.3	177.8	66.69	31.85	28.00	49.91	
**I51**	114.5	175.5	63.53	37.25	29.80/17.29	14.05	
**D52**	123.3	178.0	56.77	40.49	178.2		
**A53**	124.7	180.3	54.73	21.54			
**L54**	123.7	178.0	57.75	40.48	27.53	26.88/22.52	
**Y55**	120.2	176.8	63.29	39.17	132.6		
**F56**	118.1	178.9	61.85	38.30			
**S57**	118.2	176.6	64.38	62.42			
**V58**	122.2	179.1	67.33	31.71	23.75/21.28		

**V59**	114.1	178.2	65.13	30.21	20.15/19.07		
**T60**	120.2	175.0	67.21	67.78			
**L61**	120.7	175.5	57.75	43.22	26.16		
**T62**	98.26	176.7	61.71	69.72			
**T62'**	97.27	176.9	61.61	69.79	21.87		
**T63**	110.8	171.9	64.56	69.30			
**T63'**	110.4	173.8	64.83	68.79	21.08		
**T63''**		172.7					
**V64**	124.6	178.0	65.97				
**V64'**	128.9	178.9	67.14	32.93	22.04		
**V64''**	127.5	178.9	66.65	33.10			
**G65**	102.3	172.6	43.70				
**G65'**	106.0	173.0	43.43				
**G65''**	105.3		43.38				
**P71**		175.7	62.95	31.85	28.51	50.44	
**Q72**	121.3	178.1	55.26	31.64	34.17	180.6	
**T73**	115.6	175.4	60.40	71.65	21.63		
**D74**	125.6	179.0	57.96	39.07			
**F75**	119.1	177.9	58.77	38.24			
**G76**	108.1	178.1	46.85				
**K77**	127.4	177.6	61.44	33.20	26.92	30.08	Cε:42.61
**I78**	118.6	177.4	64.56	37.34	29.08/17.78	12.43	
**F79**	118.7	176.2	62.25	42.06			
**T80**	113.0	174.6	67.09	69.08	21.11		
**I81**	120.5	175.5	66.30	38.25	29.52/17.50	14.03	
**L82**	116.8	177.0	58.01	41.35			
**Y83**	120.8	177.1	61.97	40.17	130.4		
**I84**	117.4	177.8	65.21	38.10	30.43/16.96	16.19	
**F85**	117.1	178.1	61.00	37.55			
**I86**	119.6	177.7	65.50	36.76	29.30/17.77	13.39	
**G87**	107.9	175.5	48.41				
**I88**	121.0		64.42	37.33	29.00/17.83	12.45	

**G89**	107.8	175.5	48.17		
**L90**	121.8	179.1	58.40	41.76	26.66

The chemical shifts are referenced to DSS (external) and were deposited in the BMRB [ID: 27219 (the ion-free conformation, residues marked with a prime) and 27220 (the K^+/Rb^+-favored conformation, residues without prime)].

Supplementary Table 4 | Chemical shift assignments for SF residues of NaK under various ionic conditions.

		< 1 μM ions		50 mM Na^+			50 mM K^+		50 mM Rb^+	
		ion-free	K^+/Rb^+-favored	ion-free	K^+/Rb^+-favored	Additional conformation	ion-free	K^+/Rb^+-favored	ion-free	K^+/Rb^+-favored
**Thr62**	**N**	97.5	98.6	97.3	98.3		97.9	98.7	97.4	98.9
	**Cα**	61.64	61.74	61.61	61.71		61.56	61.74	61.76	61.99
	**CO**	176.7		176.9	176.7		176.7			176.1
**Thr63**	**N**	110.0		110.4	110.8		110.4	112.9		110.4
	**Cα**	65.25		64.83	64.56		65.07	64.67		64.96
	**CO**	173.8	172.0	173.8	171.9	172.7	173.8	171.9	173.8	172.0
**Val64**	**N**	128.9	124.4	128.9	124.6	127.5	128.9	124.4	128.9	123.2
	**Cα**	67.12	65.95	67.14	65.97	66.65	67.11	65.91	67.14	65.68
	**CO**	178.8	178.0	178.9	178.0	178.9	178.8	178.0	178.9	178.3
**Gly65**	**N**	105.7	102.3	106.0	102.3	105.3	106.0	101.4	106.0	101.9
	**Cα**	43.38	43.68	43.43	43.70	43.38	43.40	43.74	43.45	43.66
	**CO**			173.0	172.6					

Chemical shifts are referenced to DSS (external).
