# Supplementary material for: A single NaK channel conformation is not enough for non-selective ion conduction
Source: Nat Commun. 2018 Feb 19;9:717. doi: 10.1038/s41467-018-03179-y (PMC5818664; doi:10.1038/s41467-018-03179-y)
Supplement: Supplementary file 2 — Description of Additional Supplementary Files [file 41467_2018_3179_MOESM2_ESM.docx]

**Description of Additional Supplementary Files**

File Name: Supplementary Movie 1

Description: **Outward K^+^ ion permeation within a 500 ns trajectory from the computational electrophysiology MD simulations.** The simulations were performed with the crystal conformation in all subunits (simulation I, Supplementary Table 1). For clarity, only two subunits of NaK are shown. K^+^ ions are shown as red spheres. Most of the time, two K^+^ ions simultaneously occupied the S3 and S4 ion binding sites together with one ion in the vestibule.

File Name: Supplementary Movie 2

Description: **Inward K^+^ ion permeation within a 400 ns trajectory from the computational electrophysiology MD simulations**. The simulations were performed with the crystal conformation in all subunits (simulation I, Supplementary Table 1). For clarity, only two subunits of NaK are shown. K^+^ ions are shown as red spheres. Most of the time, two K^+^ ions simultaneously occupied the S3 and S4 ion binding sites together with one ion in the vestibule.

File Name: Supplementary Movie 3

Description: **Outward Na^+^ ion permeation within a 200 ns trajectory from the computational electrophysiology MD simulations.** The simulations were performed with a mixture of crystal and flipped conformations in different subunits (simulation V, Supplementary Table 1). For clarity, only two subunits of NaK are shown. Na^+^ ions are shown as blue spheres. Most of the time, only one Na^+^ ion resided in the SF.

File Name: Supplementary Movie 4

Description: **Inward Na^+^ ion permeation within a 500 ns trajectory from the computational electrophysiology MD simulations.** The simulations were performed with a mixture of crystal and flipped conformations in different subunits (simulation V, Supplementary Table 1). For clarity, only two subunits of NaK are shown. Na^+^ ions are shown as blue spheres. Most of the time, only one Na+ ion resided in the SF. Na^+^ ions entered the SF through the side entry instead of passing through the S1 and S2 ion binding sites.
